# Mid-term outcomes and hemodynamic performances of Abbott Epic mitral bioprosthesis: a single-center study

**DOI:** 10.1007/s11748-025-02212-4

**Published:** 2025-10-28

**Authors:** Takayuki Gyoten, Yu Miyama, Yu Kumagai, Yuta Kanazawa, Taiyo Kuroda, Takayuki Akatsu, Yuko Gatate, Osamu Kinoshita, Toshihisa Asakura, Akihiro Yoshitake

**Affiliations:** 1 Department of Cardiovascular Surgery, Saitama Medical University International Medical Center, 1397-1 Yamane, Hidaka, Saitama, Japan; 2 Department of Pathology, Saitama Medical University International Medical Center, Saitama, Japan

**Keywords:** Mitral valve plasty, · Replacement ·, Epic valve

## Abstract

**Objective:**

This study aimed to report “real-world” mid-term clinical experiences and outcomes after surgical mitral valve replacement with the Epic bioprosthesis in a high-volume Japanese heart center.

**Methods:**

Patients undergoing surgical mitral valve replacement with the Epic bioprosthesis from 2012 to 2023 were enrolled. Postoperative outcomes, survival, and hemodynamic performance were analyzed. The study endpoints were all-cause mortality, freedom from mitral valve reintervention, and major adverse cardiac events.

**Results:**

A total of 122 patients (mean age: 73 ± 8 years, 73 males) successfully underwent surgical mitral valve replacement with the Epic bioprosthesis (25 mm, *n* = 22; 27 mm, *n* = 37; 29 mm, *n* = 26; and 31 mm, *n* = 37). The primary valve etiologies were infective endocarditis (*n* = 17), stenosis (*n* = 18), stenosis and regurgitation (*n* = 13), regurgitation (*n* = 73), and thrombosis (*n* = 1). The median follow-up period was 33 (interquartile range: 20–48) months. Overall survival rates at 1, 3, and 5 years after mitral valve replacement were 86.8%, 82.0%, and 76.9%. The rates of freedom from rehospitalization for heart failure were 96.7% at 1 year, 95.5% at 3 years, and 91.2% at 5 years. The mean pressure gradient was 5 (interquartile range: 4–6.4) mmHg at discharge, 5.4 (interquartile range: 4.3–6.9) mmHg at 1 year, and 5.4 (interquartile range: 4–7.5) mmHg at late follow-up (Friedman test, *p* = 0.46). During the study period, only one patient required reintervention because of valve deterioration at 31 postoperative months.

**Conclusion:**

The clinical outcomes of mitral valve replacement with the Epic bioprosthesis are satisfactory, with stable hemodynamics and extremely low incidence of structural valve deterioration and reintervention over 5 years.

## Introduction

Over the last two decades, surgical mitral valve plasty (SMVP) has become a treatment option with improved outcomes for mitral valve (MV) disease [1–3]. The success of various previously reported plasty techniques is attributed to tremendous functional advances in addressing the durability and effectiveness of SMVP in the mitral complex (MV ring, papillary muscle, and chordae). However, a limited number of patients may convert to surgical mitral valve replacement (SMVR) because of unsuccessful SMVP and strong MV deterioration, including endocarditis and MV stenosis [4, 5]. Therefore, SMVR continues to be an important treatment option for these patients.

The Epic MV (Epic porcine xenograft; Abbott Cardiovascular Inc., St. Paul, MN, USA) has been implanted as a third-generation bioprosthesis. It received a CE mark in 2001 and was approved for SMVR by the FDA in 2007. Published results for SMVR with the Epic bioprosthesis in Europe and the USA suggest relatively good hemodynamic performance and acceptable rate of freedom from structural valve deterioration (SVD) [6–8]. Nonetheless, clinical studies on SMVR with the Epic bioprosthesis in a Japanese cohort are extremely scarce. Therefore, the current study aimed to report “real-world” mid-term clinical experiences and outcomes after SMVR with the Epic bioprosthesis in a high-volume Japanese heart center.

## Materials and methods

### Study design and follow-up

All patients who underwent SMVR with the Epic bioprosthesis were considered eligible for this retrospective analysis. Patients aged ≤ 18 years or undergoing concomitant left ventricular assist device implantation were excluded. This single-center study was conducted in accordance with the principles embodied in the 1964 Helsinki Declaration and all subsequent revisions and was approved by the institutional ethics committee of the Saitama Medical University International Medical Center, Japan (reference no. 2025–211). The requirement for the acquisition of informed consent from patients was waived owing to the retrospective nature of this study.

The clinical course of all patients based on their charts was retrospectively reviewed and routinely assessed before surgery, after surgery in the intensive care unit, and at discharge. Follow-up data on clinical status and transthoracic echocardiography (TTE) were obtained from the patients’ general practitioners or private cardiologists via telephone and fax communication and were complete in 100% of patients. The clinical follow-up was closed on April 31, 2025, when the last enrolled patient had completed 1-year follow-up.

Cardiac surgery, anticoagulation management, and anti-infection therapy were carried out in a routine manner [9]. TTE data were available for all patients preoperatively. All patients underwent TEE at discharge, 1 year after SMVR, and late follow-up. TTE examinations were performed by two experienced cardiologists, and all evaluations were conducted according to standard techniques recommended by the American Society of Echocardiography [10].

### Endpoints

The study endpoints were overall survival and major adverse cardiac events (MACCE). Rehospitalization for heart failure (HF) was defined as new-onset or worsening signs and symptoms of HF that required urgent therapy and resulted in hospitalization. SVD was defined as any dysfunction or deterioration involving the prosthesis (excluding infection or thrombosis), as determined by reoperation, autopsy, or clinical investigation.

### Surgical procedures

Experienced board-certified cardiovascular surgeons performed all procedures via median sternotomy or right thoracotomy. SMVR with concomitant procedures, including aortic valve replacement, tricuspid valve repair (TVR), coronary artery bypass grafting, pulmonary vein ablation, and other procedures (atrial septum defect [ASD] closure and resection of the left atrial appendage) were performed via full sternotomy, if possible. Cardiopulmonary bypass (CPB) was established through direct cannulation of the ascending aorta and right atrial vein or superior vena cava/inferior vena cava in cases requiring TVR and ASD closure. With the right thoracotomy approach, a skin incision was made in the fourth intercostal space after establishing CPB through femoral artery and vein cannulation. Access to the MV was gained via either a direct left atrial or transseptal incision, depending on the need for TVR and ASD closure. Standard MV replacement (MVR) was performed on the arrested heart at normal temperature (36 °C). As part of our standard strategy, the posterior leaflet is preserved while the anterior leaflet is resected. In cases of infective endocarditis, both leaflets are entirely resected. Intraoperative transesophageal echocardiography was performed to verify the absence of MR in SMVR. All patients were treated with warfarin as anticoagulation therapy for 3 months postoperatively.

### Statistical analysis

Results are expressed as mean ± standard deviation (SD) or median + 25th–75th percentile interquartile range (IQR) for continuous variables and as frequency and percentage for categorical variables, as indicated. Univariate comparisons were performed using Student’s paired or unpaired t-tests for normally distributed continuous data. Nonparametric continuous data were compared using the Mann–Whitney U test. The Friedman test was performed for normally distributed continuous data among the three groups. Differences were considered statistically significant at *p*-values < 0.05. All statistical analyses were performed using R software.

## Results

### Study population

A total of 122 patients (mean age: 73 ± 8 years, 73 males) underwent SMVR with the Epic bioprosthesis (25 mm, *n* = 22; 27 mm, *n* = 37; 29 mm, *n* = 26; and 31 mm, *n* = 37). Since 2019, the Epic bioprosthesis was implanted in > 80% of the enrolled patients. Furthermore, 7% of patients were aged < 60 years at the time of SMVR. The primary MV etiologies requiring surgical replacement were infective endocarditis (*n* = 17), stenosis (*n* = 18), stenosis and regurgitation (*n* = 13), regurgitation (*n* = 73), and SVD of the mechanical valve (*n* = 1). Repeat MVR was performed in 22 patients (18%). The median follow-up period was 33 (IQR: 20–48) months. The surgical approach to the MV was full sternotomy in 119 (98%) patients and right thoracotomy in 3 (2%) patients. Isolated MVR was performed in 24 patients (20%). At least one concomitant procedure, including coronary artery bypass grafting, other valve surgeries, maze procedure, or aortic surgery, was performed in 98 (80%) patients. The demographic and clinical features are summarized in Tables 1 and 2, respectively.

**Table 1:** Baseline characteristics

Characteristics	*N* = 122
Age, years	73 ± 8
Male, *n*	73
Hight, cm	159 ± 9
Weight, kg	55 ± 11
Hypertension, *n*	51 (42)
Diabetes mellitus, *n*	19 (16)
Hyperlipidemia, *n*	25 (20)
Peripheral artery disease, *n*	3 (2.5)
Hemodialysis, *n*	4 (3.3)
Prior stroke, *n*	24 (20)
Atrial fibrillation, *n*	68 (56)
Laboratory	
Aspartate aminotransferase, U/L	24 (IQR: 19–32, range: 12–120)
Alanine aminotransferase, U/L	18 (IQR: 12–25, range: 3–138)
Creatinine, mg/dL	0.97 (IQR: 0.73–1.22, range: 0.42–8.48)
Estimated glomerular filtration rate, mL/min/1.73 m^2^	53 (41–70, 5.1–122)

*n* (%) if not otherwise specified. *IQR* interquartile range

**Table 2:** Procedural characteristics

Characteristics	*N* = 122
Repeat surgery, *n*	22 (18)
Minimally invasive cardiovascular surgery, *n*	3 (2.5)
Pathology for mitral disease	
Mitral regurgitation, *n*	73 (60)
Mitral stenosis, *n*	18 (15)
Infective endocarditis, *n*	17 (14)
Mitral stenosis and regurgitation, *n*	13 (11)
Thrombosis (mechanical valve), *n*	1 (0.8)
Valve size	
25, *n*	22 (18)
27, *n*	37 (30)
29, *n*	26 (21)
31, *n*	37 (30)
Isolated mitral valve replacement, *n*	24 (20)
Concomitant procedures	
Aortic valve replacement, *n*	31 (25)
Tricuspid valve repair, *n*	59 (48)
Left atrial appendage closure, *n*	63 (52)
Coronary artery bypass grafting, *n*	16 (13)
Maze procedure, *n*	30 (25)
Ascending aorta replacement, *n*	1 (0.8)
Atrial septum defect closure, *n*	7 (5.7)
Others, *n*	3 (2.5)

*n* (%) if not otherwise specified

### Early clinical outcomes after SMVR

The overall mortality rate at 30 days was 2.5% (*n* = 2), with right HF (*n* = 1) and cardiogenic shock (*n* = 1) being the reasons for death. No valve-related operative mortality (i.e., no MV dysfunction) occurred during the perioperative period. No early reoperation, including for recurrent infective endocarditis, perivalvular leak, or valve deterioration, was performed within 1 month from the operation.

### Later clinical outcomes and survival

**Fig. 1:**
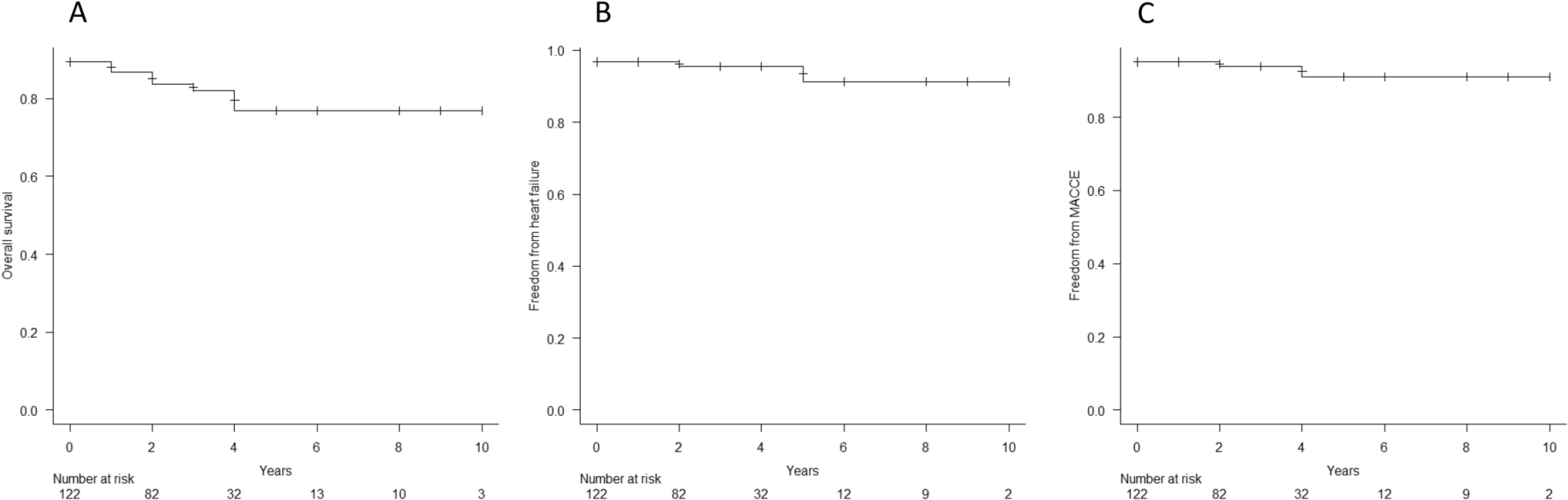
The Kaplan–Meier curve after SMVR. **a** The Kaplan–Meier overall survival rates were 86.8% (95% CI: 79.3–91.7%) at 1 year, 82.0% (95% CI: 73.2–88.1%) at 3 years, and 76.9% (95% CI: 65.3–85.0%) at 5 years. **b** The Kaplan–Meier rates of freedom from rehospitalization for heart failure were 96.7% (95% CI: 91.5–98.8%) at 1 year, 95.5% (95% CI: 89.5–98.1%) at 3 years, and 91.2% (95% CI: 76.2–96.9%) at 5 years. **c** The Kaplan–Meier rates of freedom from MACCE were 95.1% (95% CI: 89.4–97.8%) at 1 year, 93.9% (95% CI: 87.6–97.1%) at 3 years, and 91.0% (95% CI: 80.6–95.9%) at 5 years

The median follow-up period was 33 (IQR: 20–48) months. A total of 22 patients (18%) died at follow-up. The Kaplan–Meier overall survival analysis of this cohort is shown in Fig. 1.

The Kaplan–Meier overall survival rates were 86.8% (95% CI: 79.3–91.7%) at 1 year, 82.0% (95% CI: 73.2–88.1%) at 3 years, and 76.9% (95% CI: 65.3–85.0%) at 5 years after MVR (Fig. 1a). The reasons for death are summarized in Table 3.

**Table 3:** Postoperative outcomes at early- and mid-term follow-up

Characteristics	*N* = 122
Mortality at 30 days	1.6% (*n* = 2)
Late follow-up	
Reasons of death, *n*	20
Cardiac death	1
Right heart failure, *n*	1
Non-cardiac death	19
Sepsis	2
Pneumonia	4
Stroke	3
Malignancy	1
Lung bleeding	1
Old age	4
Unknown	4

The Kaplan–Meier rates of freedom from rehospitalization for HF were 96.7% (95% CI: 91.5–98.8%) at 1 year, 95.5% (95% CI: 89.5–98.1%) at 3 years, and 91.2% (95% CI: 76.2–96.9%) at 5 years after MVR (Fig. 1b). The Kaplan–Meier rates of freedom from MACCE were 95.1% (95% CI: 89.4–97.8%) at 1 year, 93.9% (95% CI: 87.6–97.1%) at 3 years, and 91.0% (95% CI: 80.6–95.9%) at 5 years after MVR (Fig. 1c).

### Valve hemodynamics

**Fig. 2:**
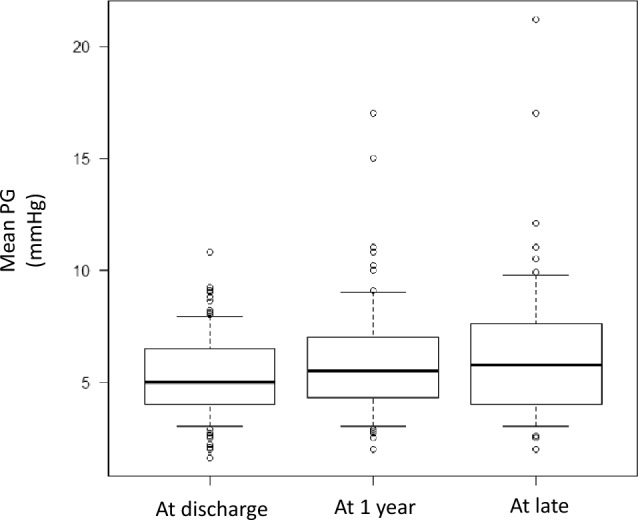
The mean mitral valve gradient by echocardiography. No statistically significant difference among the three points was found (*p* = 0.46).PG, pressure gradient

Postoperative echocardiography data were available for 121 patients (99%). The mean MV gradient did not differ significantly among three time points (5 [IQR: 4.0–6.4] mmHg at discharge vs. 5.4 [IQR: 4.3–6.9] mmHg at 1 year after SMVR vs. 5.4 [IQR: 4.0–7.5] mmHg at late follow-up after SMVR [a median of 3 years (IQR: 2–4 years, range: 2–11 years)]; *p* = 0.46) (Fig. 2a). According to the classification of the size of implanted Epic valves, the mean gradient of the 25-mm MV measured at discharge was significantly increased compared to that of the 27-, 29-, and 31-mm MV (*p* = 0.023). However, the mean MV gradient measured 1 year postoperatively did not differ among the four sizes (*p* = 0.47). This trend remained consistent at late follow-up, and the mean PG values for all sizes were within the normal range. The mean gradient for each valve size was not significantly different at discharge, 1 year, and late follow-up (Table 4).

**Table 4:** Comparison on the mean gradient for each valve size

Valve size	At discharge	At 1 year	At late follow-up	P-value
25 mm, mmHg	6.1 (IQR: 5.0–7.1)	6.0 (IQR: 5–7.2)	7.1 (IQR: 5.9–9.1)	0.93
27 mm, mmHg	5.0 (IQR: 4.0–6.0)	5.0 (IQR: 4.4–6.6)	4.7 (IQR: 3.9–7)	0.64
29 mm, mmHg	5.0 (IQR: 3.0–6.9)	5.6 (IQR: 3.7–8)	5.4 (IQR: 3.9–6.9)	0.69
31 mm, mmHg	4.0 (IQR: 4.0–5.4)	5.2 (IQR: 4.2–6.3)	5.0 (IQR: 4–6.4)	0.38

*IQR* interquartile range

### Pathology of SVD

**Fig. 3:**
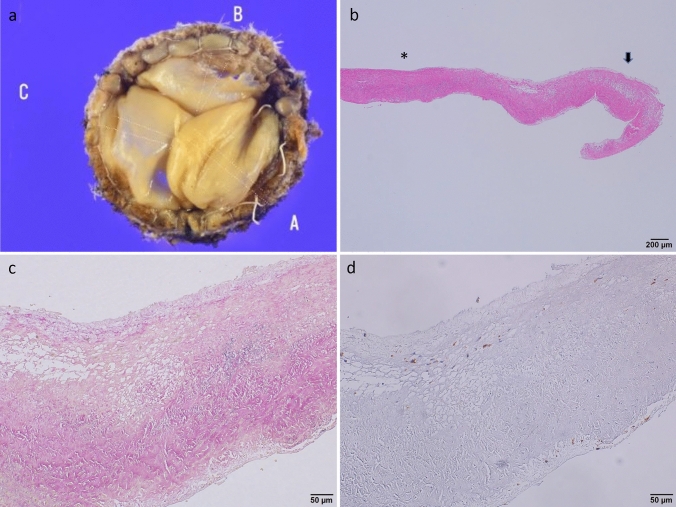
Pathological findings of the removed mitral bioprosthesis valve. **a** Degenerative changes in macro-findings were observed in only one cusp. **b** The connective tissue around the teared regions (arrow) of the degenerative cusp was loose, compared with other regions (*). (c and d) Associated loose connective tissue

During the study period, only one patient required reintervention because of valve deterioration at 31 months postoperatively. The patient successfully underwent repeat SMVR. The macro-pathological findings of the removed valve indicated that one cusp was torn with degenerative alteration but without calcification. The micro-findings of the torn tissue revealed that a number of CD68( +) macrophages were clustered on the tissue and the elastic membrane was sparse, unlike the other two cusps that maintained normal elastic tissue. Immune responses to CD3 and CD20 were negative (Fig. 3).

## Discussion

In this study, we summarized the clinical outcomes of SMVR with the Epic MV in symptomatic patients with various etiologies of MV disease. The current study yielded two main findings. First, the clinical outcomes, including overall survival, freedom from rehospitalization for HF, and MACCE, were acceptable. Second, the hemodynamics of the Epic MV were stable at 3-year (median) follow-up after MVR, and only one patient required repeat MVR.

Over the past two decades, a significant evolution in MV surgery has occurred, with repair being performed rather than replacement [1–3]. In the modern era, intervention therapy with the MitraClip (MC, Abbott Vascular, Santa Clara, CA, USA) device has been developed and is currently regarded as the first-line therapy for MR in old patients or patients with high surgical risk [11]. However, SMVR continues to be an important treatment option for patients with MV disease that is not amenable to repair (e.g., patients with recurrent MV disease after SMVP, infective endocarditis, MR after failed MitraClip therapy, and MV stenosis) [12–14]. Therefore, SMVR remains a durable option, although it may be sometimes performed as “bail-out” surgery [12].

Published reports worldwide had estimated a survival rate of 60–70% at 8–10-year follow-up, and this result was concluded as a satisfactory outcome [15, 16]. At 5 years, the rate of freedom from SVD was approximately 93–97% [16, 17]. A recent nationwide study reported that the rates of freedom from all-cause valve reintervention and freedom from rehospitalization for HF were > 90% and > 50%, respectively, at 10 years after MVR with the Epic MV [18]. In our cohort, SMVR with concomitant procedures was performed in 80% of the enrolled patients. The mortality rate during the perioperative period was as high as 2.5%; however, the rate of freedom from HF and MACCE at 5 years after SMVR was > 90%, and this clinical outcome seems satisfactory.

We used Epic MVs of various sizes, among which 31-mm MV was often implanted. Because the etiology of MV disease was MR, the MV annulus was secondarily dilated, and a much larger MV was used. Interestingly, the mean pressure gradient in the MV at 1 year after SMVR and at late follow-up was not significantly different. In addition, we found no patients requiring reintervention due to endocarditis, thrombosis, or stenosis, implying that SMVR with the Epic bioprosthesis results in satisfactory hemodynamics at 1 month after SMVR. This trend remains the same at mid-term follow-up, even with small valve sizes (25 mm). Further accumulation of cases and evidence, including mid-to long-term results, is required.

In our study, only one patient required surgical reintervention for SVD occurring at 3 years after SMVR. The removed product showed that one cusp in the Epic MV was torn, resulting in severe MR. Pathological findings indicated that macrophages clustered on the torn cusp and the elastic membrane was spared, different from the other two cusps. Interestingly, the Epic MV comprised three cusps with valves from at least two pigs. To our understanding, this biological reaction of CD68-positive macrophages seems to be an immune reaction against one cusp as “foreign material” [19]. Although the SVD of the bioprosthesis valve is already known under the concept of a lipid-mediated inflammatory mechanism, a large-scale study with pathological examination is required to identify this concept. Further studies dealing with pathological evidence should be performed in the future.

In our opinion, the number of SMVR will increase owing to the increased number of interventional therapies using the MitraClip device. SMVR is necessary in cases of failed MitraClip therapy, sometimes as “bail-out surgery” [12]. In addition, younger patients have recently received bioprosthesis owing to the interventional valve in valve therapy as a less invasive treatment [20]. Improved outcomes of valve therapy in the mitral position may boost the utility of bioprostheses.

Our study has strength in that it is one of the few reports on the Epic MV used in a Japanese cohort. Nonetheless, this study has several limitations. This was a retrospective single-center study that included a limited number of patients. Although this study did not allow for generalized statements, we were able to report good outcomes in this cohort.

## Conclusion

The clinical outcomes and hemodynamics after SMVR with the Epic MV in the Japanese population are satisfactory. SVD is an uncommon indication for reintervention.

## Data Availability

The data underlying this article are available.
